# Unholy marriages and eternal triangles: how competition in the mushroom life cycle can lead to genomic conflict

**DOI:** 10.1098/rstb.2015.0533

**Published:** 2016-10-19

**Authors:** Sabine Vreeburg, Kristiina Nygren, Duur K. Aanen

**Affiliations:** Laboratory of Genetics, Plant Sciences Group, Wageningen University, 6700 AA Wageningen, The Netherlands

**Keywords:** fungi, life cycle, sex, genetic conflict, basidiomycetes, mating system

## Abstract

In the vast majority of sexual life cycles, fusion between single-celled gametes is directly followed by nuclear fusion, leading to a diploid zygote and a lifelong commitment between two haploid genomes. Mushroom-forming basidiomycetes differ in two key respects. First, the multicellular haploid mating partners are fertilized in their entirety, each cell being a gamete that simultaneously can behave as a female, i.e. contributing the cytoplasm to a zygote by accepting nuclei, and a male gamete, i.e. only donating nuclei to the zygote. Second, after gamete union, the two haploid genomes remain separate so that the main vegetative stage, the dikaryon, has two haploid nuclei per cell. Only when the dikaryon produces mushrooms, do the nuclei fuse to enter a short diploid stage, immediately followed by meiosis and haploid spore formation. So in basidiomycetes, gamete fusion and genome mixing (sex) are separated in time. The ‘living apart together’ of nuclei in the dikaryon maintains some autonomy for nuclei to engage in a relationship with a different nucleus. We show that competition among the two nuclei of the dikaryon for such ‘extramarital affairs’ may lead to genomic conflict by favouring genes beneficial at the level of the nucleus, but deleterious at that of the dikaryon.

This article is part of the themed issue ‘Weird sex: the underappreciated diversity of sexual reproduction’.

## 1. Introduction

Sex (see box 1 for our definition of sex) starts with fusion between gametes, which brings the genomes of different organisms together in a single zygote and thereby introduces competition between homologous genes for transmission to offspring. Furthermore, during sexual reproduction, an individual transmits its genome not as a single entity but as different fractions, i.e. different sets of genes that replicate together (referred to as co-replicons [2]), which can be subject to different transmission rules. For example, in organisms with genetic sex determination, sex chromosomes are unevenly transferred to males and females, in contrast to the autosomes. Similarly, cytoplasmic genes, such as mitochondrial or chloroplast genomes, usually are passed on via the female lineage only. These different transmission rules imply that different co-replicons within an individual can have different fitness optima, leading to conflict between them. So, in this respect, sex has two consequences: (i) it introduces competition between homologues within a single co-replicon category, and (ii) it introduces conflict between the genes of different co-replicons due to different transmission rules. It is important here to emphasize the difference between competition and conflict. Competition occurs between entities within the same category, such as between homologous genes, or between different mitochondrial genomes. Essentially, competition implies wanting the same. By contrast, there can only be conflict between entities of different categories within an individual, such as between the nuclear and mitochondrial genomes, or between non-homologous genes within a nuclear genome. Essentially, therefore, conflict means wanting something different, such as ‘meiotic drive’ versus ‘fair meiosis’, or ‘male sterility’ versus ‘male fertility’.

Box 1. **The definition of sex.**

There are many (evolutionary) biology or genetics textbooks in which sex is being discussed, but no explicit definition of sex is given; it is apparently generally assumed that the reader knows what sex is. However, the implicit definitions of sex tend to be narrow, not applicable to all classes of organisms, e.g. involving the fusion of two different gametes like an egg and a sperm cell. As in the example, characteristics of a species' life cycle are often included in the definition, thereby neglecting other species that are considered to have sex but have different life cycles. This caveat was also pointed out by Dick [1], who consequently defined sexual reproduction as ‘the union of two haploid nuclei each derived from one of two different meioses. The advantage of Dick's definition of sex is that it allows for separation of the universal nuclear events, i.e. the effect of sex on the genome, from the typical life cycle events that can differ extensively between classes of organisms. We do think, however, that Dick's definition is incomplete in one aspect, namely the subsequent reduction of fusion product's genome by meiosis. Hence, our definition of sex is the union of two haploid nuclei, each produced by meiosis, in due course followed by a reduction of the genome through meiosis. According to this definition, sex in basidiomycetes is separated in time from gamete fusion, which is in contrast to almost all other sexual life cycles.

Both consequences of sex stated previously provide opportunities for selfish (or ultra-selfish [3]) genes, i.e. genes that decrease the fitness of the individual carrying them and, therefore, depend on other means to increase in frequency, to compensate for the harm they incur on their host. Such compensation can occur through horizontal spread to other individuals or through violation of a transmission ‘rule’ such as meiosis, leading to meiotic drive. Ultimately, ultra-selfish genes can be selected due to intra-individual competition between homologous genes. Since ultra-selfish genes by definition decrease individual fitness, they will be in conflict with all other unlinked genes in the genome and will thus lead to genomic conflict [4].

Sexually reproducing organisms have evolved various lifecycle adaptations to reduce the selective scope for ultra-selfish genes. For example, uniparental transmission of cytoplasmic genes reduces the selective scope for ultra-selfish mitochondrial and chloroplast genes, as the fates of the cytoplasmic genes and the maternal lineage become united [2,5]. Also, the union of the two haploid nuclei into a single diploid nucleus, followed by mitotic divisions of that nucleus, and a ‘fair’ meiosis in the sexual organs removes the opportunity for the haploid nuclear components to pursue their own selfish interests by, for example, outcompeting the other by faster replication. Yet, certain sexually reproducing organism groups are lacking some of these adaptations to reduce the selective scope for ultra-selfish genes. This begs the question whether such organisms are more prone to genomic conflicts due to ultra-selfish genes. In this article, we consider this question for filamentous basidiomycete fungi in which we can distinguish two categories of co-replicons based on their transmission during mating, *viz*. nuclei and mitochondria. (In this review, we assume that meiosis is ‘fair’, so we do not consider the possibility of meiotic drive, which has been found to play a role in some ascomycete fungi [6].)

**Figure 1.**
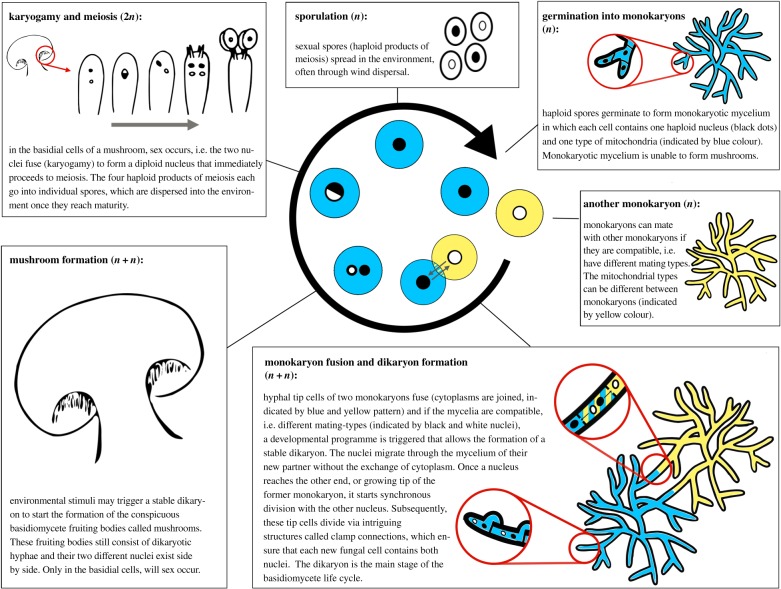
The standard life cycle of mushroom-forming basidiomycetes is usually based on *Schizophyllum commune*, one of the best studied species of this group. This species is obligatorily outcrossing, a reproductive system called **heterothallism**. In this life cycle, haploid spores germinate to form a **monokaryon**, a sterile hyphal network called **mycelium**, in which each cell contains a single haploid nucleus. Two monokaryons can fuse and if the mycelia are compatible, i.e. if they have different **mating types** (genetically defined sexual compatibility traits), a developmental programme is triggered that leads to the formation of a stable **dikaryon**, a mycelium of which the cells contain two haploid nuclei. The nuclei migrate through the mycelium of their new partner without the exchange of cytoplasm and mitochondria. Once a migrating nucleus reaches the growing tip of the receiving monokaryon, it starts synchronous division with the other nucleus. From now on, these tip cells divide via an intriguing structure called a **clamp connection**, which ensures that each new fungal cell contains both nuclei. In most species, the dikaryon can live for many years and increase in size by mitotic divisions. Certain environmental stimuli can trigger the formation of the sexual fruiting bodies called **mushrooms**. These fruiting bodies mostly consist of dikaryotic hyphae. Only in specialized cells called **basidia**, does sex occur, i.e. the two nuclei fuse to form a temporary diploid nucleus that immediately proceeds to meiosis. The four haploid products of meiosis each go into individual spores, which are dispersed in the environment once they reach maturity. In **homothallic** species, selfing occurs, so that a single sexual spore on its own can form fruiting bodies and basidiospores.

Filamentous basidiomycete fungi differ in several key aspects from the most well-known sexual organisms, such as animals, plants and ascomycete fungi (figure 1). First, gamete fusion does not occur during the single-celled stage, but between **monokaryons** (all terms printed in bold are explained in the legend of figure 1), multicellular haploid **mycelia**, which mate in a hermaphroditic fashion. Two cells of the different monokaryons fuse creating one cell where both nuclei and cytoplasms are shared. Subsequently, if the two nuclei are compatible, i.e. if they have different **mating types**, both monokaryons allow their partner's nuclei to migrate through their mycelium until they reach the other side of the former monokaryon (now **dikaryon**; figure 1). Thus, barring the narrow zone of interaction where the cells fuse, there is no exchange of mitochondria. Second, unlike the vast majority of sexual organisms, upon fertilization, the nuclei do not fuse, but remain separate in each cell of the dikaryon. It is only in specific cells, the **basidia**, of the **mushrooms** that the two nuclei will fuse, just before meiosis (figure 1). Consequently, sex in basidiomycetes is separated in time from gamete fusion, which is in contrast to almost all other sexual life cycles. Third, although the dikaryon cannot be fertilized by a second haploid nucleus, it retains the potential to donate nuclei to another monokaryon [7,8]. We systematically explore the potential for genomic conflict in this life cycle and discuss empirical evidence for the theoretical predictions.

## 2. The monokaryon: gametes and sexual roles

What are the male and female gametes in the basidiomycete life cycle [9]? The answer to this question is important, since it is one of the determinants for genomic conflict. In the standard life cycle of basidiomycetes, two monokaryons reciprocally fertilize each other by simultaneously donating nuclei to, and receiving nuclei from, their mating partner (figure 1). Ultimately, the donated nuclei are the male gametes as these fertilize the monokaryon without prior investment in growth. It is less obvious what the female gamete is. As each monokaryon can be fertilized in its entirety, the monokaryon can be considered as a single female ‘super gamete’. Alternatively, as each cell of the monokaryon can be fertilized, the monokaryon can also be considered as a sexual organ and each of its cells as a female gamete. This is not just a theoretical argument, since a single monokaryon can be fertilized by multiple genetically different nuclei, from multiple monokaryons [10]. However, since the monokaryon exhibits both a female and male role, i.e. the reception and donation of nuclei, respectively, and can grow vegetatively, the monokaryon also is a multicellular individual, albeit a special one, since each cell can become fertilized. So in essence, each cell of the monokaryon can behave as a female gamete and each nucleus as a male gamete.

### (a) The female role: exposed to risks and bearing the costs

Although the monokaryon needs to accept another nucleus to become fertilized and reproduce sexually, by doing so it exposes itself to risks and potential costs. At ‘gamete fusion’, the nucleus of the receiving monokaryon is providing a soma to the nucleus of its mating partner, while its partner provides a nucleus only. If the resulting dikaryon is poorly functioning, the cost is borne by the receiving monokaryon, which contributed the cytoplasm, and which now has decreased or even lost its chance to reproduce. One example would be the invasion of selfish nuclei, which might take over the cytoplasm and eliminate the original nucleus. An observation in spore-trap experiments indicates that this might happen in *Schizophyllum commune* [11]. Also, in the genus *Armillaria*, it has been shown that there can be hostile takeovers of one nucleus by a different one, although this is an exceptional genus as it has diploid nuclei [12,13]. Similarly, in pairings between selfing and outcrossing populations of the species *Stereum hirsutum*, replacement of the nucleus of the outcrossing population by a nucleus of the selfing population has been demonstrated [14]. Even without selfish elements, the new nucleus might carry genes that are not well adapted to the environment or not compatible with the original nucleus. This may render the dikaryon less adapted to the environment than the original monokaryon. Thus, this ‘female role’ of the monokaryon is believed to be one chance only, since the accepted nucleus cannot be aborted, even if the resulting dikaryon is maladapted.

Furthermore, although the dikaryon resulting from a mating between monokaryons cannot accept another nucleus, it can still donate one of its nuclei to a monokaryon [7,15], a phenomenon called the ‘Buller phenomenon’ or ‘di-mon mating’ [8]. However, by accepting another nucleus, and forming a dikaryon, the receiving nucleus enters into competition with the nucleus it just accepted for future success in di-mon matings.

So there are two potential costs of accepting a nucleus. The first arises from accepting a nucleus that results in a maladapted dikaryon. The second cost arises from accepting a more competitive nucleus that wins in the competition for di-mon fertilizations. Both costs should promote the evolution of female choice [10,16].

## 3. Recognition of the sources of male gametes and its consequences

The male role of a monokaryon is the donation of nuclei to its mating partner. However, there are also other sources of male gametes. First, sexual spores can act as male gametes if they land on an established monokaryon [9,11,17]. Second, as described above, the dikaryon resulting from a mating between monokaryons can still donate one of its nuclei to a monokaryon [7,15], the so-called ‘Buller phenomenon’ [8]. The recognition of the various sources of male gametes has important implications for the opportunities for sexual selection, genomic conflict and the calculation of the cost of sex.

### (a) Outcrossing basidiomycetes have a male-biased operational sex ratio

Since the dikaryon cannot receive another nucleus, a di-mon mating is equivalent to a mating between a male and a female. Because of this, all populations of outcrossing basidiomycetes, which consist of ‘male’ dikaryons and ‘hermaphroditic’ monokaryons, have a male-biased operational sex ratio, which can increase the importance of sexual selection [10,16] (see also §5b).

### (b) The one-and-a-half-fold cost of sex

**Figure 2.**
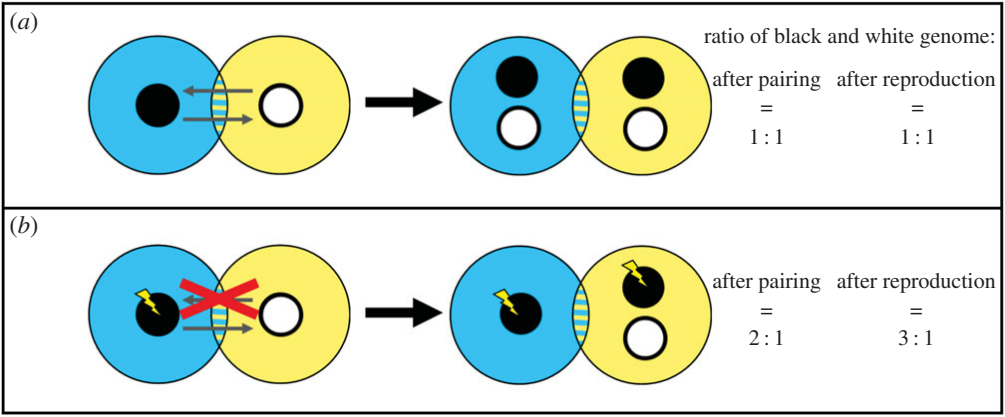
(*a*) Schematic illustration of a pairing between two monokaryons, where each monokaryon dilutes its genetic constitution by the acceptance of a nucleus and compensates for the dilution by simultaneously donating a nucleus to its partner monokaryon. (*b*) As in *a*, with the difference that one of the monokaryons has gained a mutation that prevents dilution of its genetic constitution, by inducing asexual reproduction or sexual reproduction via selfing, while still being able to donate a nucleus. While the ratio of black and white genome after pairing has changed from 1 : 1 to 2 : 1, the ratio of black and white genome in the spores has changed from 1 : 1 to 3 : 1, assuming that both parts of the mycelium produce equal numbers of spores.

The hermaphroditic nature of pairings between two monokaryons can provide the conditions for the selection of ultra-selfish genes. As explained above, in the regular life cycle of mushroom-forming basidiomycetes, the monokaryon simultaneously donates and accepts another nucleus. By accepting another nucleus, a monokaryon dilutes its own genetic constitution, but compensates for this genome dilution by donating a nucleus to its partner monokaryon. Imagine a mutation in a monokaryon that prevented its genomic dilution by prohibiting the acceptance of another nucleus and enabled asexual reproduction or selfing, while still allowing the fertilization of another monokaryon (figure 2). Everything else being equal, the benefit of this mutation would be 50% relative to its wild-type allele [18]. Since this benefit is shared with all other genes of that nucleus, there is no genomic conflict at the level of the monokaryon (see [18] for a more extensive discussion).

#### (i) Changing life cycles

A similar argument can be made for the initial benefit of another, similar kind of mutation occurring in a dikaryon of an obligatorily outcrossing (so-called **heterothallic**—a mating system where mating can only occur between gametes with different mating types) species. If this mutation changes the outcrossing life cycle into a selfing (**homothallic** or secondarily homothallic—a mating system where mating can occur between gametes with identical mating types) or asexual life cycle, all spores produced by this dikaryon contain the mutation. If such a mutant still allows the dikaryon to donate one of its nuclei to a monokaryon, it theoretically gains a benefit over the wild type. The exact magnitude of this benefit is hard to calculate since it depends on the frequency of di-mon matings in a population, which we do not know. The example of *S. hirsutum* discussed above, in which a nucleus of a selfing population is shown to have been donated to monokaryons from an outcrossing population, illustrates that such mutations do exist [14].

*Agaricus bisporus* is one of the mushroom-forming basidiomycetes in which an outcrossing mating system evolved into a (partially) selfing (secondarily homothallic) mating system [19]. In this species, there are two inter-fertile varieties that both produce dikaryotic and monokaryotic spores, but in a different ratio. In *A. bisporus* var*. bisporus* (all present-day commercial varieties and most wild varieties), two meiotic products, i.e. two nuclei, are packaged into one spore in over 90% of the basidia in such a way that both parental mating types are represented in each spore [20–23]. As both parental mating types are present in these dikaryotic spores, they are self-fertile. By contrast, *A. bisporus* var. *burnettii* (a subspecies isolated from the Californian desert) predominantly produces four monokaryotic spores, which are, therefore, not self-fertile [20,22].

As explained above, the transition from obligate outcrossing (heterothallism) could be explained by an initial fitness gain of the selfing (homothallic) variety in an outcrossing (heterothallic) population [18]. However, this predicted fitness gain might be severely reduced as the new life cycle will lead to inbreeding and increased homozygosity. Yet, this particular species appears to have evolved a way to minimize the effects of inbreeding by redirecting meiotic crossovers almost exclusively to its chromosome ends [22,24]. By having crossovers at chromosome ends, the offspring of these mushrooms have practically intact parental chromosomes. Combined with predominantly non-sister nuclei pairing in spores, this ensures that approximately 90% of the var. *bisporus* spores almost only differ from their parental genotypes by a reshuffling of homologous chromosomes over the two nuclei. All chromosomes combined still form the same genome. Hence, genetically, the resulting mushrooms are almost identical to the parental mushroom, which limits the detrimental effects of inbreeding.

## 4. The dikaryon: genomic conflict due to mitochondrial competition

**Figure 3.**
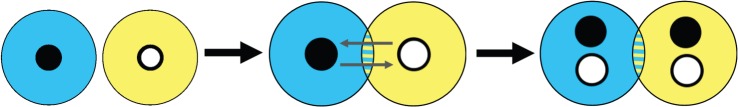
Schematic representation of a mating between two monokaryons. In a mating between two monokaryons, both nuclei (one white, one black) migrate to fertilize their respective partner mycelium. The cytoplasms, and thus the mitochondria (blue and yellow), are not exchanged, except for in the narrow interaction zone in which the hyphal tips fuse (blue and yellow stripes). Consequently, a single dikaryon is formed in which all cells have the same nuclear genotype, but may have different mitochondrial genotypes.

During a mating between two monokaryons, a combination of migration and nuclear division transforms the two monokaryons into a single dikaryon in terms of nuclear genomes. All cells of the dikaryon contain two nuclei, one of each monokaryon. By contrast, the mitochondria of each monokaryon do not migrate, so that the two mitochondrial types of the resulting dikaryon are separated in space (figure 3). This gives rise to possible competition among the mitochondrial haplotypes from the two original monokaryons, within the newly formed dikaryon.

Since most cells of the dikaryon contain only a single type of mitochondria, and each cell potentially can give rise to a mushroom, cytoplasmic inheritance is doubly uniparental: both monokaryons involved in a mating can potentially transmit their cytoplasm to the sexual spores, but normally only a single type per spore [25]. In this life cycle, within-cell competition between genetically different mitochondria is limited, since the only cells that contain the two types of mitochondria are the fused cells at the initiation of mating (figure 1). However, at the dikaryon level, the two types of mitochondria do compete over transmission. This is a peculiar situation: although there is restricted cytoplasmic exchange, there is nevertheless enduring physical contact between cells with two types of mitochondria. If individual mitochondria can increase their relative chance to be included in the spores, and if this occurs at a cost of dikaryon fitness, this leads to genomic conflict for two reasons:

(i) a mitochondrial gene can be selected at the level of the cytoplasmic genome but selected against at the level of the dikaryon and,
(ii) because nuclei are homogeneously distributed in the dikaryon, nuclear fitness is directly dependent on dikaryon fitness. A reduction in dikaryon fitness because of intra-dikaryon mitochondrial competition is therefore directly in conflict with nuclear interests.

One theoretical possibility for ultra-selfish behaviour of mitochondrial genes is via the induction of male sterility. A monokaryon normally both accepts its partner's nucleus and donates its own, which are female and male roles, respectively. Theoretically, a mitochondrion that can prevent the male role of the monokaryon it resides in while maintaining its female role (cytoplasmic male sterility or CMS) will have a selective advantage over a partner mitochondrion that does not do so for two reasons (which are not mutually exclusive):

(i) such a mitochondrion will monopolize the spores, because fruiting in the other section of the mycelium will be prevented and,
(ii) in most basidiomycetes, the relative growth rate of a dikaryon is higher than that of a monokaryon [26,27]. Therefore, even postponing male function relative to female function can be advantageous for an individual mitochondrion.

Aanen *et al.* [25] have modelled the evolution of mtDNA-induced male sterility. In their model, there were male-sterile and male-fertile mtDNAs and nuclear determinants specifying either resistance or susceptibility to CMS. In a monokaryon with a resistant nucleus, the effect of mtDNA-based male sterility is nullified, and nuclear migration occurs irrespective of whether the other monokaryon is male sterile or male fertile. The model explained data obtained for the genus *Hebeloma* reasonably well [25].

In plants, CMS is well established and leads to gynodioecy, a mating system with female and hermaphroditic plants (cf. [28,29]). In all described cases, CMS is encoded by mitochondrial mutations, while resistance genes exist in the nuclear genome. For basidiomycetes, the question is what possibilities mitochondria would have to induce male sterility. A theoretical mechanism for male sterility is that mtDNA mutations somehow block the mating pheromone receptors of the other monokaryon or block the production of the pheromones of the male-sterile strain. Also, if a mitochondrion can induce the dikaryon to produce more mushrooms in the part of the mycelia with that mitochondrion, it will increase its proportion of spores, even without causing male sterility. However, it remains to be demonstrated that CMS plays a general role in basidiomycete fungi.

## 5. The dikaryon—nuclei living apart together: genomic conflict due to nuclear competition

### (a) Nuclear competition during vegetative growth and asexual reproduction

Another source of genomic conflict arises from competition among the nuclei within the dikaryon during vegetative growth and asexual propagation [30,31]. If the replication of the nuclei in the mycelium is not regulated, one nucleus can divide faster than the other, increasing its relative abundance within the mycelium, even if this decreases the fitness of the mycelium. A study on vegetative growth rates of monokaryons and dikaryons of *Heterobasidion parviporum* confirmed that it is possible for a nucleus to dominate the dikaryon, even when it leads to a decreased growth rate [32].

Alternatively, a nucleus can be opportunistic in positioning itself towards the hyphal tip. Because growth in filamentous fungi occurs at the edge of the mycelium, those nuclei that position themselves at the hyphal tips take part in growth and can replicate [33]. In most ascomycetes and some basidiomycetes, mitotic growth is not well regulated [34]. These fungi can form multinucleate heterokaryotic cells, in which the ratios between the two types of nuclei can deviate strongly from 50–50 [32,35,36]. This can lead to the escape of monokaryotic hyphae (e.g. *A. bisporus* [37]) and to the production of monokaryotic asexual spores (oidia) favouring the nuclear type that is in the majority (e.g. *Heterobasidion annosum*, *H. parviporum*, *Pholiota microspora* (*Pholiota nameko*) [32,36,38–40]). By contrast, in *Termitomyces*, another basidiomycete fungus with multiple nuclei per cell, no monokaryotic escapes have been found [41]. Similarly, in some of the mutualistic fungi cultivated by fungus-growing ants, multiple nuclei are present in each cell. However, in contrast to *Termitomyces*, more than two haploid genomes are maintained within a single mycelium, although at present it is unknown whether those genomes are distributed *between* different haploid nuclei or *within* polyploid nuclei [42].

In most basidiomycete species, the formation of so-called **clamp connections** between dikaryotic cells prohibits deviations from a one-to-one ratio among the two nuclear types during somatic growth of the dikaryon (figure 1). Nevertheless, if monokaryotic spores are formed during asexual spore production, one nuclear type could still be over-represented in species with clamp connections. It is striking though that the two nuclei of a dikaryon cell appear to change position after each conjugate division [43]. Although asexual reproduction probably is less important in most species of basidiomycetes than in ascomycetes, it is tempting to consider this highly regulated change in position of the nuclei as a specific ‘policing’ mechanism at the level of the dikaryon to reduce the risk that one nuclear type can monopolize asexual monokaryotic spores. Nevertheless, the two possible kinds of asexual monokaryotic spores formed by a dikaryon with clamp connections can show strong deviations from a 50–50 ratio [36,44]. If the gene that biases the nuclear ratio comes at a cost for the total number of spores produced by the dikaryon, there is genomic conflict.

### (b) Nuclear competition during Buller interactions

In §2a, we predicted that a monokaryon will be choosy in accepting a nucleus, since the receiving nucleus will enter into competition with the accepted nucleus for future success in Buller pairings. In this section, we address the potential implications for genomic conflict of this within-dikaryon inter-nuclear competition for di-mon matings [7]. Since most populations of basidiomycetes contain hundreds of mating types, the vast majority of nuclei are compatible. So both nuclei of a dikaryon usually will be compatible with an unrelated monokaryon and will compete to fertilize it in a di-mon interaction. If there would be no difference between the two nuclei, each would have 50% chance to fertilize the monokaryon. However, it has been found that the success of nuclei often strongly and consistently deviates from a one-to-one ratio in favour of one of the two, even if both are compatible with the monokaryon [16,45–47]. In theory, such success in Buller matings could be due to a mutation (or multiple mutations) that increases the success of its containing nucleus even if that same mutation reduces dikaryon fitness as a pleiotropic effect. This is a conflict between a gene selected at the level of the nucleus in competition with another nucleus, and all other nuclear genes, which depend on dikaryon fitness (except for half of the genes residing in the same nucleus as the mutation, but only until recombination cuts that linkage). But how large can this conflict be?

**Figure 4.**
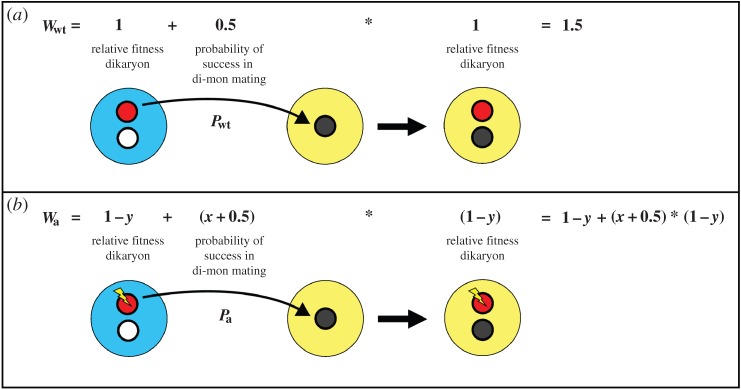
Schematic illustration of a di-mon pairing, where the two nuclei of a dikaryon compete to fertilize a monokaryon.



Let a mutation *a* give a nucleus a benefit *x* in competition with a nucleus with the wild-type allele, so that its success in Buller matings is 0.5 + *x* (0 ≤ *x* ≤ 0.5) relative to the wild type, but it decreases the relative fitness (*w*) of the dikaryon it finds itself in with *y*, so (figure 4):

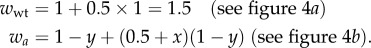


If

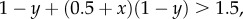
*a* will be selected.

Now we consider the maximum possible value of *x* (0.5) to calculate the maximum fitness reduction *y* for the mutation still to be selected:

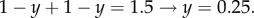


So the maximum tolerable fitness reduction for a mutation that increases the success in Buller pairings to 100% is 0.25.

The above calculation ignores at least two important complications. First, the selection of an ultra-selfish gene is frequency dependent. So, to calculate its fitness, we would need to consider the change in frequency during selection to find its equilibrium frequency. Second, assuming that mildly ultra-selfish genes may go to fixation, they will form the starting situation for a possible new round of selection for other ultra-selfish mutations. So, the maximum value calculated above only applies to one particular time point and for one possible kind of interaction. However, the major force determining the selective advantage of a mutation leading to an increased male performance in di-mon matings is the—unknown—frequency of di-mon mating events in nature. Using experimental evolution in *S. commune* for increased male fertility, increased male mating success could be selected, but generally no clear trade-offs were found with other fitness components, such as growth rate [48].

### (c) Possible mechanisms for winning the competition between nuclei

#### (i) Mating types

Basidiomycetes generally have many mating types, up to thousands [49]. In most basidiomycetes, the mating type is determined by two loci: a locus encoding a homeodomain transcription factor (HD), the ‘A’ locus, and a locus encoding pheromones and pheromone receptors (P/R), the ‘B’ locus (see [50] for an excellent overview). Two nuclei are compatible if the alleles at both mating-type loci are different; therefore, the probability of being compatible is mainly determined by the number of mating types at the locus with the lowest number of variants. The mating-type genes have been found to evolve faster than genes involved in other conserved functions [51,52], and especially at the P/R locus, there is a large variety of pheromone alleles. It has most often been inferred that the enormous diversity in mating types allows for maximum outbreeding, while it reduces sibling compatibility. Almost any randomly encountered individual will have a different mating type, whereas only 25% of the progeny of the same fruiting body will be compatible [53]. However, this hypothesis does not account for the high degree of redundancy, i.e. usually multiple compatible pheromone–receptor interactions are found between alleles, whereas a single compatible interaction is sufficient. Therefore, Nieuwenhuis and Aanen [10] proposed the alternative hypothesis that the mating-type genes are a target of sexual selection in basidiomycete fungi (see also [54]). More specifically, they hypothesized that the redundancy in pheromones at the P/R locus is a consequence of sexual selection [10]. As explained above, in di-mon matings, competition can arise between the two nuclei of a dikaryon that might be costly to the dikaryon as a whole. It would be interesting to test in future studies (i) if the P/R mating type locus is indeed responsible for the observed deviation in success between the two nuclei of a dikaryon in a Buller mating and (ii) if the locus itself, or any linked genes, have a negative effect on overall dikaryon fitness.

#### (ii) Nucleus-specific and parent-of-origin effects on gene expression

Since there is no genetic sex determination in basidiomycetes, potential conflicts could be combatted by differential gene regulation in a way analogous to genomic imprinting (the differential expression of alleles of a gene depending on its parent of origin) found in mammals and plants [55,56]. However, with the marked difference that, whereas in other organisms the imprint is determined by the sex of the meiotic parent, in basidiomycetes the difference in gene regulation would have to depend on the sex role taken by the monokaryon at fertilization.

In di-mon matings, the two male gamete types, i.e. the nuclei of the dikaryon, compete for the fertilization of the monokaryon. This male–male competition is analogous to sperm competition, with the fundamental difference that it occurs within the cell in which the two competitors are together and generally assumed to cooperate. Potentially, one nucleus could regulate gene expression in the other nucleus in such a way that it reduced the male potential of its competitor nucleus. In particular, the receiving monokaryon might have most power to suppress the future male role of the fertilizing nucleus, since the receiving nucleus contributes the cytoplasm to the initial dikaryon. Given that the haploid monokaryotic mycelium does not need to form a dikaryon for survival and vegetative growth, the hypothetical extreme case scenario is that the second nucleus is active only during sexual reproduction. This putative mechanism would remove all competition in Buller matings, while leaving the outcrossing advantage of sexual reproduction intact.

A study of the ascomycete fungus *Neurospora tetrasperma* has shown that gene expression levels can differ between the two nuclei in a dikaryon [57]. Unlike most ascomycetes, the vegetative mycelium of *N. tetrasperma* commonly consists of two nuclear types, but the ratio of the two nuclei was found to deviate from one to one. However, the relative gene expression of a few investigated genes did not reflect the ratio of each nuclear component in the mycelium [57]. Although this example is an ascomycete species, it shows that the relative gene expression of a nucleus does not necessarily correlate with its frequency in the mycelium.

The differences in gene expression between the two nuclei are not necessarily the result of competitive interaction. If one nucleus is better adapted to the environment, the dikaryon as a whole could benefit if that nucleus became dominant, in terms of gene expression. Under such a scenario, differential gene expression would be adaptive for both nuclei. Alternatively, differences in gene expression between nuclei are the result of competition. Recently, research has shown that selfish behaviour of nuclear variants within the mycelium of the acomycete *Neurospora crassa* can occur. In an evolution experiment, cheater nuclei were selected that had a relative benefit in competition with wild-type nuclei, at the cost of the total number of spores produced by the mycelium [58]. However, the mechanism by which these differences in competitive success are achieved remains to be explored.

## 6. General discussion

The specific aspects of the sexual life cycle of mushroom-forming basidiomycetes leave room for the selection of ultra-selfish genetic elements that are in conflict with the rest of the genome. This implies that basidiomycete ‘organismality’, i.e. the extent to which the parts composing a social group, in this case a multicellular individual, work together for the common whole, is lower than in other multicellular organisms [59]. A corollary of this is that organismal fitness may be suboptimal and that differentiation is less irreversible than in animals and plants.

By contrast to a diploid organism with a single fused, diploid nucleus and one type of mitochondrial genome (and other cytoplasmic organelles) per cell, the dikaryon consists of multiple genetic entities that form an ‘unholy marriage’ as they can still pursue their own selfish interest to some degree, even if this comes at a cost to the dikaryon as a whole. The two genetically different haploid nuclei of the dikaryon remain separate until just before meiosis and the dikaryon might also be a mosaic of mitochondrial types, although most cells will contain only a single type. So sex in basidiomycetes is separated in time from gamete fusion, and the nuclei remain separate for most of the vegetative stage until just before meiosis, facilitating ‘eternal triangles’ during the dikaryon stage. Furthermore, gametes in the basidiomycete life cycle are not single cells, but multicellular organisms that mate in a hermaphroditic fashion.

There is an interesting parallel between basidiomycetes and mosses. In mosses, a haploid gametophyte produces eggs that get fertilized by sperm produced by the same or a different gametophyte to produce a diploid sporophyte. Also, after fertilization, the gametophyte ‘mother’ supports that diploid sporophyte, which grows on top of her [60]. Similar to the monokaryon in basidiomycetes, in the moss life cycle, the haploid ‘mother’ thus invests before fertilization and supports an unrelated haploid genome of her mating partner after fertilization. In both life cycles, conflicts between male and female haploid 'interests’ may be played out both before and after gamete fusion.

The first main category of possible genomic conflicts we identified is between mitochondrial and nuclear DNA, due to the different inheritance modes of these genomes. A possible result of this type of conflict is the evolution of mitochondrion-induced male cytoplasmic sterility. However, there is not much evidence that this type of conflict is important, and it seems hard to imagine that the mitochondrial DNA has sufficient possibilities to induce male sterility in the basidiomycete life cycle.

The second main source of genomic conflicts is nuclear competition. We have pointed out that a monokaryon can simultaneously behave as a male (each nucleus) and female gamete (each monokaryotic cell). Once fertilized, the resultant dikaryon has lost its female potential, but retains its male potential via so-called di-mon (or Buller) pairings. The consequential male-biased operational sex ratio, combined with the potential cost of accepting a nucleus, could lead to selection for ultra-selfish mutations, providing a benefit to a nucleus in competition with the other nucleus. This process can also be interpreted in terms of sexual selection. The one theoretical prediction is that the nuclei in the dikaryon are in competition to fertilize monokaryons, which may favour traits that provide a selective benefit at the level of the nucleus, but that are harmful for the dikaryon. We have calculated the maximum tolerable costs for such mutations using some simplifying assumptions and have shown that these costs can be significant. The other prediction is that the monokaryon will be under selection to be critical to the nucleus she accepts, as this determines her fitness. A possible mechanism for winning the competition among nuclei is via the pheromones, encoded by one of the mating-type loci, and we have argued that the extreme redundancy of pheromones compared with pheromone receptors that is observed may be a consequence of this selection process.

Another consequence of the ‘living apart together’ of the two haploid nuclei in the dikaryon is that competition occurs to enter the asexual spores. Even though asexual reproduction is less important in basidiomycetes than in many other fungi, there are some examples where one nucleus has a higher representation in the asexual spores.

A different type of ultra-selfish mutations is a mutation that monopolizes the spores. A mutation that turns a monokaryon or a dikaryon into asexual or selfing (homothallic) reproduction, while retaining the possibility to donate nuclei, would be selected. This selective benefit could be the explanation for frequent changes in life cycles seen in some basidiomycete groups, such as the genera *Agaricus*, *Coprinus* and *Mycena*.

The analysis in this paper is largely theoretical. Most research on competition and conflict in the mushroom life cycle has been in laboratory settings with only a small set of model organisms. We see two main lines of progress for future research. First, experimental evolution may provide the experimental tests of theoretical predictions, and some progress has recently been made (cf. [48,61]). Second, due to progress in genetic and genomic techniques, the possibilities of studying natural populations and non-model organisms will increase (cf. [62]). These recent advances will allow us to assess the relevance of the potential sources of genomic conflict in the basidiomycete life cycle that we have identified in this paper.
